# Independent and Joint Contributions of Fine Particulate Matter Exposure and Population Vulnerability to Mortality in the Detroit Metropolitan Area

**DOI:** 10.3390/ijerph15061209

**Published:** 2018-06-08

**Authors:** Amy J. Schulz, Graciela B. Mentz, Natalie Sampson, Melanie Ward, J. Timothy Dvonch, Ricardo de Majo, Barbara A. Israel, Angela G. Reyes, Donele Wilkins

**Affiliations:** 1 Department of Health Behavior and Health Education, University of Michigan School of Public Health, Ann Arbor, MI 48109, USA; gmentz@umich.edu (G.B.M.); melaward@umich.edu (M.W.); rdemajo@umich.edu (R.d.M.); ilanais@umich.edu (B.A.I.); 2 Department of Health and Human Services, University of Michigan-Dearborn, Dearborn, MI 48128, USA; nsampson@umich.edu; 3 Department of Environmental Health Sciences, University of Michigan School of Public Health, Ann Arbor, MI 48109, USA; dvonch@umich.edu; 4 Detroit Hispanic Development Corporation, Detroit, MI 48216, USA; agreyes@dhdc1.org; 5 Green Door Initiative, Detroit, MI 48213, USA; donele@greendoorinitiative.org

**Keywords:** fine particulate matter, cardiopulmonary risk, population vulnerability, cumulative risk

## Abstract

Fine particulate matter is associated with adverse health outcomes. Exposure to fine particulate matter may disproportionately affect urban communities with larger numbers of vulnerable residents. We used multilevel logistic regression models to estimate the joint effects of fine particulate matter (PM_2.5_) and population vulnerabilities on cardiopulmonary mortality (CPM). We estimated the health benefits of reductions in PM_2.5_ across census tracts in the Detroit metropolitan area with varying levels of population vulnerability, using cluster-specific odds ratios scaled to reflect PM_2.5_-attributable cardiopulmonary risk. PM_2.5_ and population vulnerability were independently associated with odds of CPM. Odds of CPM and the number of deaths attributable to PM_2.5_ were greatest in census tracts with both high PM_2.5_ exposures and population vulnerability. Reducing PM_2.5_ in census tracts with high PM_2.5_ would lead to an estimated 18% annual reduction in PM_2.5_-attributable CPM. Between 78–79% of those reductions in CPM would occur within census tracts with high population vulnerabilities. These health benefits of reductions in PM_2.5_ occurred at levels below current U.S. reference concentrations. Focusing efforts to reduce PM_2.5_ in the Detroit metropolitan area in census tracts with currently high levels would also lead to greater benefits for residents of census tracts with high population vulnerabilities.

## 1. Introduction

Exposure to ambient air pollution is associated with increased risk of multiple adverse health outcomes, including cardiovascular mortality [1,2,3], respiratory hospitalization [4], asthma exacerbation [5,6,7], incidence and duration of respiratory symptoms [8,9], declines in lung function [10,11,12], preterm delivery and low birth weights [13,14,15], and restricted activity [16,17]. Evidence suggests that fine particulate matter (PM_2.5_) is more strongly associated with morbidity and mortality than coarse particulate matter (PM_10_) [18], with adverse health effects observed at pollution levels below current U.S. National Ambient Air Quality Standards (NAAQS) and common to many U.S. cities [19,20].

There is substantial evidence that non-Hispanic Black (NHB) and Hispanic communities, and those with low to moderate incomes, are disproportionately exposed to air pollutants [21,22,23,24]. NHBs and Hispanics are also more likely to reside in urban communities with reduced access to quality education and employment opportunities [25,26]. A sizeable literature documents increased vulnerability of residents of low to moderate income urban communities and communities with greater race-based residential segregation to air pollution and associated health risks [21,27,28,29]. Low to moderate economic status contributes to challenges avoiding exposure and may exacerbate adverse health effects of exposures [30] through mechanisms including, for example, poorer quality housing [31,32] or reduced access to health care [33]. Children and older adults are more susceptible to adverse health effects of exposure [34,35,36,37,38]. Yet, relatively few studies have examined combined effects of exposure to air pollution in conjunction with population vulnerabilities, or quantified the health impacts associated with reductions in air pollution in communities as these may vary with characteristics of exposed populations. Using data from the Detroit metropolitan area (DMA), we examine the independent and joint contributions of air pollution exposure and population vulnerabilities to cardiopulmonary mortality and estimate the number of deaths averted by focusing efforts on places with varying constellations of characteristics.

**Figure 1:**
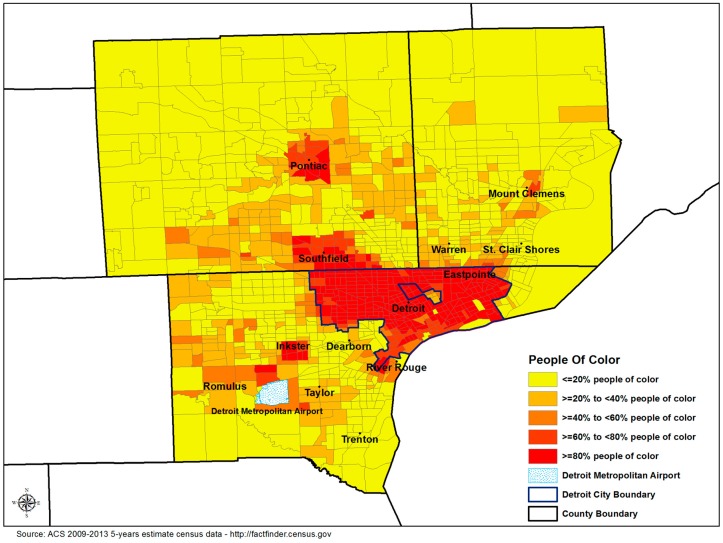
Proportion of persons of color at the census tract level, Detroit metropolitan area.

The Detroit metropolitan area (DMA), comprised of Macomb, Oakland, and Wayne Counties, is among the most racially segregated regions in the nation [39]. Historically, numerous factors contributed to this segregation, including restrictive agreements prominent prior to 1948 that prohibited racial and ethnic groups from living in parts of the city of Detroit [40]. Continued discrimination in the real estate market, including federally-backed home mortgages available to non-Hispanic Whites (NHWs) but not NHBs, perpetuated segregation and reduced NHBs’ access to suburban employment opportunities [40,41]. Today’s DMA reflects these historical and contemporary processes (see Figure 1), with NHB populations in Wayne, Oakland, and Macomb counties clustered in urban communities [42]. Eighty-five percent of Wayne County’s NHB population is located in Detroit city [42], also home to an Hispanic community whose roots extend back nearly a century [43]. NHBs and Hispanics across the DMA are disproportionately represented in households below the federal poverty line [44]. Over 57% of Detroit’s children [45,46], compared to 37%, 20%, and 13% of children in Wayne, Macomb, and Oakland counties, respectively, live in households below the poverty line [42].

Segregation contributes to health inequities through economic divestment, limited educational and employment opportunities, as well as foreclosure risk in racially segregated urban communities [34,40,47,48,49].

Segregation may also contribute to unequal exposure to air pollution. For example, in the DMA, census tracts with greater concentrations of NHB and Hispanic residents, households below the poverty line, and residents who have not completed high school are more likely to have higher levels of exposure to diesel PM (a component of PM_2.5_) and associated cancer and respiratory health risks [22]. This study extends previous research by examining the independent and joint effects attributable to fine particulate matter (PM_2.5_) and population vulnerabilities on three health outcomes previously found to be associated with air pollution: mortality attributable to ischemic heart disease [1,2], cardiovascular disease [1,50], and cardiopulmonary disease [3]. In addition, it quantifies the health impacts of reductions in PM_2.5_ in communities with high and low population vulnerability scores.

## 2. Materials and Methods

The study was conducted as part of Community Action to Promote Healthy Environments, a community-based participatory research partnership designed to equitably engage community, academic, and health service provider organizations in research, and aid the translation of results from that research into action to promote health equity (see Acknowledgments). The University of Michigan granted institutional review board approval for this study on 24 January 2013.

### 2.1. Data

Data for the analyses described below were drawn from multiple sources, informed by methods developed by a California-based team [30,51]. Information on fine particulate matter was drawn from the EPA’s National-Scale Fused Air Quality Surfaces Using Downscaling Tool and the Community Multiscale Air Quality (CMAQ) model, for the calendar year 2013. The downscaler model combines air quality monitoring and modeling data to provide better fine-scale predictions of air pollutant levels at local and community scales, and is downloadable from the EPA website [52,53,54]. Demographic data were drawn from the 2009–2013 American Community Survey [55]. Mortality data were provided by the Michigan Department of Health and Human Services (MDHHS) and include records of all deaths that occurred in the Detroit metropolitan area (Wayne, Oakland, and Macomb Counties) in 2013.

### 2.2. Measures

Dependent variables include ischemic heart disease mortality, cardiovascular mortality, and cardiopulmonary mortality, coded using the following ICD-10 codes: Ischemic heart disease, I20–I25 [56]; cardiovascular, I10–I70 [56]; and cardiopulmonary, I10–I70 and J00–J99 [56]. Mortality was coded as a dichotomous measure (i.e., ‘1’ if cause of death was ischemic heart disease, cardiovascular disease, or cardiopulmonary disease, respectively), and geocoded to individuals’ residential addresses.

Census tract level-independent variables include census tract PM_2.5_ concentrations and social, economic, and age-related vulnerabilities [30,51]. A base map was constructed that indicates residential areas and land uses where sensitive populations may be present (e.g., child care facilities, health care facilities, schools). Strictly industrial or commercial areas are not included. Areas (polygons) are roughly the size of a city block. The base map geographically links residential and sensitive land use polygons with census tract-level metrics in each of the categories of exposure and population vulnerability described below, to create a risk score for each polygon. As described below, the risk score is used to rank neighborhoods according to their level of risk (e.g., PM_2.5_ concentration, population vulnerability) across categories. Polygons are aggregated to the census tract level for this analysis.

The PM_2.5_ concentration measure was calculated at the census tract level, using annual averages. Measures were weighted by the proportion of the census tract that was residential or sensitive land use. Scores were calculated based on quintile distribution rankings, ranging from 1 (low) to 5 (high), and applied to each tract in the study area.

The vulnerability index includes the following indicators associated with increased vulnerability to environmental exposures, calculated at the census tract level [27,29,30,51,57]: percent of households below poverty; median home value (reverse coded); percent of homes occupied by renters; percent of population aged >24 years with a less than high-school-level of education (<high school); linguistic isolation (percent of residents who live in households with no adults who speak English); percent people of color; percent of population aged <5 years; and percent population aged ≥60 years. Census tracts were rank-ordered based on their scores for each of the above items and scored as quintiles from 1 (low) to 5 (high). The scores for each item were then summed, using equal weights, to create a cumulative vulnerability index reflecting population vulnerability across all included dimensions at the census tract level. Census tracts were once again rank-ordered based on these cumulative scores and divided into quintiles to create the vulnerability index score, ranging from 1 (low) to 5 (high).

Individual level control variables include age (continuous); gender (male = referent); race/ethnicity (NHB, Hispanic, NHW (referent)); education (<high school, high school graduation, >high school (referent)); marital status (not currently married (referent)); and whether the cause of death was coded on the death certificate as related to tobacco use (yes, likely, no (referent)).

### 2.3. Analysis

We used multilevel, multivariate, longitudinal analyses with indicators of mortality for 2013. The use of hierarchical linear models accounts for the longitudinal nature of the data (e.g., mortality over a one-year period), accommodating changes over time in mortality patterns and providing unbiased estimates of exposure effects. Separate models were run using ischemic heart disease, cardiovascular, and cardiopulmonary disease mortality as dependent variables. Predictor variables were grand mean centered, the logit link was used, and an exchangeable correlation matrix was used to estimate standard errors.

Random intercept only multilevel logistic models were used to assess the independent and joint associations between PM_2.5_ exposure and the vulnerability index at the census tract level with each indicator of mortality at the individual level. Models examine dynamic exposure–response relationships between exposures and each dependent variable (e.g., cardiopulmonary mortality). For each dependent variable, models examined effects of PM_2.5_ exposure and vulnerabilities, each alone, and the joint effects of the two components when included together in a model, controlling for age, gender, race, and ethnicity. We also ran models with PM_2.5_ concentration and each individual indicator included in the vulnerability index (e.g., percent of households below the poverty line). In testing joint effects, we considered multiplicative models assessing interactions between PM_2.5_ and vulnerability. Interaction terms were not statistically significant (*p* < 0.05), and thus the final models reported in this paper do not include interaction terms. To assess the sensitivity of models to varying specifications of the variables, we ran models using the rank-ordered quintile versions of each variable as described above and using continuous versions of each variable. Patterns were similar: findings are reported using the rank-ordered quintile versions, with statistical significance reported as *p* < 0.05, *p* < 0.01, and *p* < 0.001.

Finally, we estimated the health impact of reductions in PM_2.5_ in the DMA on mortality rates attributable to this exposure. To do this, we converted the original five-point scale for each index to a dichotomous variable: census tracts falling in quintiles 1–2 were collapsed and coded as “low” and those in quintiles 3–5 were collapsed and coded as “high” census tracts. We calculated odds ratios for each level (high, low) using standard statistical methods [58,59], then calculated odds ratios for each of four resulting clusters—combinations of PM_2.5_ and population vulnerability (Cluster 1 = low/low; Cluster 2 = low/high; Cluster 3 = high/low; and Cluster 4 = high/high)—by multiplying odds ratios for their respective components. Odds ratios were then scaled to reflect PM_2.5_-attributable cardiopulmonary mortality risk. Based on estimates previously reported in the literature and recognizing geospatial variations in attributable risk, we estimated reductions in cardiopulmonary mortality using 3%, 5%, 10%, and 15% PM_2.5_-attributable risk [16,58,59,60,61]. Resulting odds ratios were multiplied by the total population in respective clusters to calculate the number of deaths due to ischemic heart disease, cardiovascular disease, and cardiopulmonary disease that would be averted under each of the above scenarios. All analyses were performed using HLM 7.03 for Windows (HLM 7.03 Scientific Software International (SSI), Stokie, IL, USA).

## 3. Results

Table 1 shows descriptive statistics for each of the indicators included in the CI. Mean PM_2.5_ for census tracts in the DMA is 9.6 µg/m^3^ (SD = 0.3). For indicators of population vulnerability, on average at the census tracts level, about two-fifths of the population were of color, one in ten households had incomes below the poverty line, and about one-third of households were occupied by renters. Also, at the census tract level, on average, one in eight residents had less than a high school education level and about one in twenty residents were linguistically isolated. The average census tract had about one in 15 residents under the age of five and about one in five aged 60 or older. At the census tract level, on average, about one-fifth of deaths were attributable to ischemic heart disease, about one-third to cardiovascular disease, and just over four in 10 were attributable to cardiopulmonary causes.

Table 2 presents results from analyses examining the first research question: what are the associations between PM_2.5_ and population vulnerability and each of the three mortality indicators? Odds ratios were significantly greater for those residing in census tracts with the higher exposure quintiles. Specifically, as shown in Model 1, likelihood of death due to ischemic heart disease was significantly greater in census tracts ranked in the third (*p* = 0.01), fourth (*p* = 0.004), and fifth (*p* < 0.001) quintiles compared to the first. Likelihood of death due to cardiovascular disease was significantly greater for those in census tracts in the fifth quintile (*p* = 0.01), and deaths attributed to cardiopulmonary disease were more likely in the third (*p* = 0.07), fourth (*p* < 0.001), and fifth (*p* < 0.001) quintiles compared to those in the lowest exposure quintile.

Similarly, as shown in Table 2, in Model 2, odds ratios of ischemic heart disease were significantly higher for those living in census tracts in the third (*p* = 0.05), fourth (*p* = 0.01), and fifth (*p* < 0.001) quintiles of population vulnerability, compared to those in the lowest vulnerability census tracts. For cardiovascular disease, likelihood of mortality was significantly higher for those in the second (*p* = 0.04), third (*p* < 0.001), fourth (*p* < 0.001), and fifth (*p* = 0.01) quintiles of vulnerability; and likelihood of mortality due to cardiopulmonary disease was significantly higher in the third (*p* < 0.001), fourth (*p* < 0.001), and fifth (*p* < 0.001) quintiles of population vulnerability.

Because census tracts with greater population vulnerability scores have been previously demonstrated to have higher air pollutant exposures [22], our third research question assessed joint contributions of PM_2.5_ exposure and population vulnerabilities. Results are shown in Table 3. After accounting for population vulnerabilities, effects of PM_2.5_ on mortality remained significantly higher for ischemic heart disease in the third (*p* = 0.02), fourth (*p* = 0.03), and fifth (*p* < 0.001) quintiles of PM_2.5_ exposure, and for cardiopulmonary disease in census tracts, in the fourth (*p* = 0.03) and fifth (*p* = 0.01) quintiles of PM_2.5_ exposure.

Similarly, after including PM_2.5_ in the models, odds ratios for cardiovascular disease remained significantly higher in the second (*p* = 0.04), third (*p* = 0.001), and fourth (*p* = 0.001) and approached significance in the fifth (*p* = 0.07) quintiles of vulnerability, and for cardiopulmonary disease, in the third (*p* < 0.001), fourth (*p* < 0.001), and fifth (*p* < 0.001) quintiles of vulnerability, compared with census tracts with the lowest vulnerability scores. Models using continuous versions of PM_2.5_ and the population vulnerability index yielded similar results (see Supplementary Materials Table S1). Models testing for potential modifications of effects of PM_2.5_ by population vulnerabilities found no significant interactions for any of our three dependent variables (results not shown).

Table 4 shows the population of the DMA living in census tracts within each of four clusters: low population vulnerability/low PM_2.5_; low population vulnerability/high PM_2.5_; high population vulnerability/low PM_2.5_; and high population vulnerability/high PM_2.5_ concentration. An estimated 1,659,342 (39%) residents of the Detroit metropolitan area live in census tracts with both high PM_2.5_ concentrations and high vulnerability, with 50% (2400 of 4800) of estimated total cardiopulmonary mortality occurring in census tracts with those characteristics (Table 4). We calculated PM_2.5_-attributable scaled odds ratios for each of the four clusters. Results from tests of contrasts indicate that residents of census tracts with high vulnerability and high PM_2.5_ concentrations experience significantly greater probability of cardiopulmonary mortality compared with residents of the other three clusters (*p* < 0.001) (full calculations are shown in Supplementary Materials Table S2).

Also shown in Table 4, reductions in PM_2.5_ in census tracts with high PM_2.5_ concentrations to levels currently experienced in the low concentration census tracts would avert an estimated 15 to 75 PM_2.5_-attributable cardiopulmonary deaths each year in census tracts with high population vulnerability. An additional 4–20 PM_2.5_-attributable cardiopulmonary deaths would be averted by reducing PM_2.5_ in census tracts with high PM_2.5_ concentration and low vulnerability. This smaller number is due both to the lower probability of cardiopulmonary mortality and the lower population in census tracts with these characteristics. Overall, these estimates suggest that reducing PM_2.5_ to the levels encountered in the low exposure census tracts would avert between 19 and 95 cardiopulmonary deaths each year, approximately 13.5% of total cardiopulmonary mortality attributable to PM_2.5_ in the DMA (between 144 and 721, as shown in Table 4). (See Supplementary Materials Table S2 for greater detail on this analysis.)

**Figure 2:**
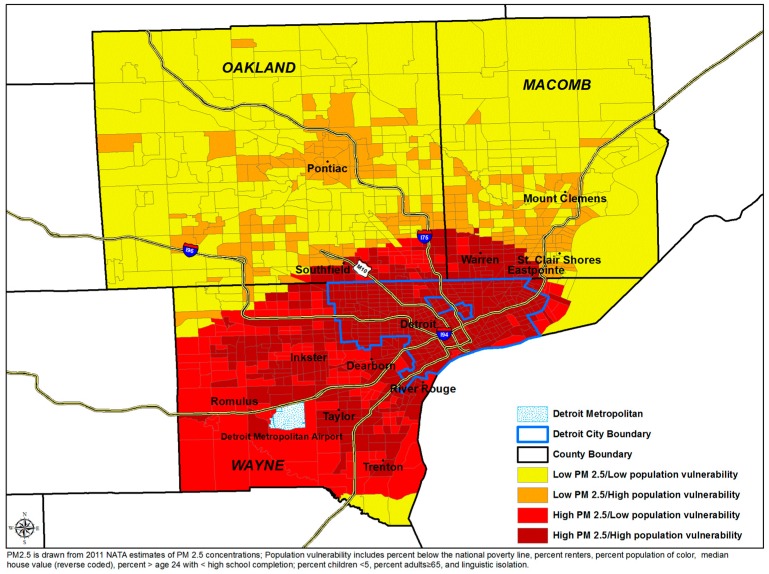
PM_2.5_ mapped at the census tract level, Detroit metropolitan area.

Census tracts with high PM_2.5_ concentration and population vulnerability are concentrated within and adjacent to Detroit city, Dearborn, River Rouge, Southfield, Inkster, Taylor, and Trenton. Based on estimates presented in Table 4, approximately 79% of the averted PM_2.5_-attributable cardiopulmonary mortality would occur in census tracts with high levels of population vulnerability, and with 40% of the population (See Figure 2).

## 4. Discussion

Findings reported here indicate that, when included in models controlling for individual demographic characteristics, census tract level indicators of exposure to PM_2.5_ air pollution and indicators of population vulnerability are associated with increased likelihood of death attributable to ischemic, cardiovascular, and cardiopulmonary causes in the DMA. Consistent with findings previously reported in the literature [62,63,64], associations of PM_2.5_ and mortality remained significant after accounting for population vulnerability (e.g., census tract socioeconomic status, racial composition). While not statistically significant, our finding suggesting that adverse effects of PM_2.5_ on mortality may be heightened in neighborhoods with higher levels of population vulnerability is consistent with results reported elsewhere [63,65,66].

Estimated reductions in mortality that would be realized with reductions in PM_2.5_ suggest that 79% of cardiopulmonary deaths averted would occur in census tracts with high levels of population vulnerability. These results add to a limited literature examining differential health impacts of reductions in air pollution by neighborhood indicators of population vulnerability. In 2014, Kheirbek and colleagues estimated health benefits associated with reductions in PM_2.5_ associated with cooking fuels in New York City, and reported the greatest benefits in high poverty neighborhoods [67]. Similarly, in 2011, Fann and colleagues [66] reported that air pollution reduction strategies that focus in areas with more susceptible/vulnerable populations yield greater reductions in mortality and in risk inequality compared with more traditional approaches used in regulatory frameworks.

These results have implications for efforts to reduce health inequities linked to unequal environmental exposures. Detroit’s industrial history, combined with historical and contemporary patterns of segregation and economic divestment from predominantly NHB and Hispanic urban communities, have contributed to both disproportionate exposure and vulnerability for NHB and Hispanic urban communities in the DMA [22]. Census tracts with both high PM_2.5_ exposure and high population vulnerability tend to be clustered in and adjacent to major urban communities, including areas with major industrial sources located in the southern, southwestern, and central areas of the Detroit metropolitan area. Effects are visible at levels of PM_2.5_ that are below the current National Ambient Air Quality Standards (NAAQS) level of 12 µg/m^3^ [68] (see Table 1), and at levels common in many urban communities in the U.S. Focusing efforts to reduce PM_2.5_ pollution in the high exposure census tracts would not only reduce population mortality, but contribute to reductions in racial, ethnic, and socioeconomic health inequities. Such efforts could include, for example, reductions in mobile sources of PM_2.5_ (e.g., reducing diesel traffic through urban residential neighborhoods); reductions in industrial pollutants (e.g., conversion to clean sources of power such as wind and solar); increases in green infrastructure, such as spatial or vegetative buffers between, for example, residential neighborhoods and heavily trafficked freeways [69,70]; or permitting for air pollution emissions that accounts for cumulative risks [70].

These analyses join similar studies [67] that illustrate the potential for using health impact assessments to inform policy and planning decisions. Nearly 40% of DMA residents live in census tracts with high vulnerability and high PM_2.5_ exposure. Residents of these census tracts experience higher probability of cardiopulmonary mortality compared with residents of census tracts with all other combinations of exposure and vulnerability. Assessments of differential health impacts of reductions in PM_2.5_ in census tracts with differential population vulnerabilities can help inform regulatory and land use decisions by incorporating analysis of implications for population health and health equity. These findings join a substantial literature indicating that particulate matter is associated with mortality (and morbidity), and that exposure to it disproportionately impacts the health of vulnerable populations [71,72]. The finding of the significant impacts of PM_2.5_ exposure on mortality despite a fairly narrow distribution of average PM_2.5_ exposure suggests the importance of continued attention to the composition as well as mass levels of PM_2.5_ [73].

### 4.1. Limitations

There are several limitations associated with this study. The models reported here examine exposure and mortality over a single year. They are neither able to examine the impacts of long-term exposures over the life course nor to disentangle the effects of exposure at particularly vulnerable periods (e.g., prenatal development, early life, puberty). The focus on mortality omits substantial contributions of both PM_2.5_ and social and economic vulnerabilities to morbidity throughout the life course (e.g., asthma exacerbations, missed work days). Finally, the relatively brief period of time covered does not allow us to disentangle, for example, the extent to which individuals with greater health challenges may move to areas with higher exposure risks and lower socioeconomic indicators due to, for example, loss of income through inability to work.

### 4.2. Strengths

These analyses also have a number of strengths. The statistical models used enable us to separate the effects of neighborhood or environmental exposures from individual characteristics, and to assess the independent contributions of PM_2.5_ exposure and population vulnerabilities at the census tract level. The use of multilevel models combined with the spatial scale of the PM_2.5_-level data (census tract level) allows us to examine variations at a relatively fine spatial scale. We are also able to estimate the number of deaths that could be averted by focusing efforts to reduce PM_2.5_ in areas with high versus low population vulnerabilities, providing valuable information to aid in decision-making processes. The use of analytical models that examine independent and joint effects of social, economic, and environmental exposures on health contributes to the ability to examine the implications of environmental rulemaking, policy, and planning decisions, and to inform those decisions by quantifying their health implications.

## 5. Conclusions

We find significant effects of environmental exposures and population vulnerabilities, assessed at the census tract level, on the likelihood of mortality due to ischemic heart, cardiovascular, and cardiopulmonary disease in the Detroit metropolitan area. Disentangling the effects of vulnerability and exposure allowed us to determine that this excess mortality disproportionately affects residents of communities with high vulnerability scores, with the greatest benefits realized within those same communities with reductions in PM exposure.

These findings extend a critical body of evidence documenting not only excess environmental exposures, but also more substantial health risks associated with those exposures in vulnerable communities, including communities of color and those with lower socioeconomic status. Combined with previously reported research demonstrating that communities with higher vulnerability experience heightened exposure to PM_2.5_, these results support the critical importance of a national research agenda focused on examining cumulative exposure and associated health risks [38,74,75], and more importantly, on the application of findings to focus efforts to reduce exposures in areas with the most vulnerable populations.

Recognizing the limitations and uncertainties associated with the current science of cumulative risk assessment, continued efforts to develop the science and apply it to analyses of risk are critical for identifying communities that experience disproportionate risk. Paying particular attention to reducing environmental exposures in communities already overburdened with multiple risks, combined with efforts to reduce the social and economic vulnerabilities experienced within those communities, stand to make important contributions to reductions in health inequities.

## Figures and Tables

**Table 1:** Descriptive statistics for individual and census tract level indicators, Detroit metropolitan area.

Individual Level (*n* = 171,000)	Percent	Mean (SD)	Range
Demographics			
Age		72.6 (18.7)	(0.0, 99.2)
Gender (Female = 1)	51.2		
Race/ethnicity			
Hispanic	1.4		
Non-Hispanic White	71.9		
Non-Hispanic Black	25.6		
Non-Hispanic Other	1.1		
Education Attainment			
Less than high school	17.3		
High school	29.1		
More than high school	17.2		
Not reported	36.4		
Married	33.6		
Smoking behavior			
Yes	7.4		
No	36.1		
Probable	5.7		
Not reported	50.8		
Mortality rates			
Ischemic heart	19.3		
Cardiovascular	34.3		
Cardiopulmonary	42.7		
**Tract Level (*n* = 1166)**			
Pollution Exposure			
PM_2.5_ (µg/m^3^)		9.6 (0.3)	(8.9, 10.0)
Vulnerability (mean percent at the tract level)			
Percent people of color		40.3 (35.4)	(0.0, 100)
Percent households living below poverty line		19.9 (16.9)	(0.0, 100)
Median home value (in thousands)		121.5 (87.1)	(10.0, 761.3)
Percent renter-occupied housing		30.9 (20.9)	(0.0, 100)
Percent aged ≥24 with <high school diploma		13.7 (10.2)	(0.0, 61.7)
Percent linguistically isolated		0.4 (1.1)	(0.0, 9.7)
Percent aged <5		5.8 (2.7)	(0.0, 17.8)
Percent aged ≥60		19.9 (6.8)	(0.0, 57.5)

**Table 2:** Mortality due to ischemic heart disease, cardiovascular disease, and cardiopulmonary disease, respectively, regressed on PM_2.5_ and cumulative vulnerability, adjusted for individual characteristics ^1^.

Tract Level Predictors	Ischemic Health Disease		Cardiovascular		Cardiopulmonary	
	Model 1	Model 2	Model 1	Model 2	Model 1	Model 2
PM_2.5_ (1 = low)	OR ^2^ (95% CI)	OR (95% CI)	OR (95% CI)	OR (95% CI)	OR (95% CI)	OR (95% CI)
2	1.06 (0.95, 1.19)		1.00 (0.92, 1.08)		0.98 (0.90, 1.07)	
3	1.17 (1.05, 1.31) ^b^		1.03 (0.95, 1.12)		1.08 (0.99, 1.18) ^†^	
4	1.19 (1.06, 1.34) ^b^		1.08 (0.99, 1.18)		1.18 (1.08, 1.29) ^c^	
5	1.31 (1.16, 1.48) ^c^		1.14 (1.03, 1.26) ^b^		1.22 (1.11, 1.34) ^c^	
Vulnerability (1 = low)						
2		1.06 (0.94,1.19)		1.10 (1.01, 1.19) ^a^		1.09 (1.00, 1.19)
3		1.12 (1.00,1.26) ^a^		1.16 (1.07, 1.26) ^c^		1.19 (1.09, 1.29) ^c^
4		1.17 (1.04,1.31) ^b^		1.17 (1.07, 1.27) ^c^		1.22 (1.12, 1.33) ^c^
5		1.24 (1.09,1.42) ^c^		1.16 (1.05, 1.29) ^b^		1.23 (1.11, 1.35) ^c^

^1^ Models were adjusted by age, gender, race/ethnicity (NHB, Hispanic, NHW), educational attainment (less than high school, high school, more than high school), death attributable to smoking (yes, probably, no), and marital status. ^a^ *p* < 0.05. ^b^ *p* < 0.01, ^c^ *p* < 0.001. ^†^ *p* = 0.07. ^2^ OR= Odds Ratio.

**Table 3:** Mortality due to ischemic heart disease, cardiovascular disease, and cardiopulmonary disease regressed on PM_2.5_ and population vulnerability at the census tract level, adjusted for individual demographic characteristics ^1^.

Quintile	Ischemic Heart		
	Disease	Cardiovascular	Cardiopulmonary
	OR (95% CI)	OR (95% CI)	OR (95% CI)
PM_2.5_ (1 = low, 5 = high)			
1	ref	ref	ref
2	1.04 (0.92, 1.17)	0.95 (0.87, 1.04)	0.94 (0.86, 1.03)
3	1.15 (1.02, 1.29) ^a^	0.99 (0.91, 1.08)	1.03 (0.94, 1.13)
4	1.15 (1.02, 1.31) ^a^	1.02 (0.93, 1.13)	1.11 (1.01, 1.23) ^a^
5	1.24 (1.08, 1.42) ^c^	1.10 (0.98, 1.22)	1.15 (1.04, 1.28) ^b^
Vulnerability (1 = low, 5 = high)			
1	ref	ref	ref
2	1.03 (0.91, 1.16)	1.10 (1.00, 1.20) ^a^	1.08 (0.98, 1.18)
3	1.07 (0.95, 1.21)	1.16 (1.06, 1.27) ^c^	1.17 (1.07, 1.28) ^c^
4	1.09 (0.96, 1.24)	1.15 (1.05, 1.26) ^c^	1.19 (1.08, 1.30) ^c^
5	1.13 (0.98, 1.31)	1.11 (0.99, 1.24) ^†^	1.15 (1.03, 1.28) ^b^

^1^ Adjusted by age, gender (ref = female), race, and ethnicity (NHB, Hispanic, NHW), educational attainment (less then high school, high school (ref) and more than high school), mortality linked to smoking (yes, probable, no (ref)) and marital status. Ranks for PM_2.5_: 1 = (0.892–0.944); 2 = (0.945–0.964); 3 = (0.965–9.74); 4 = (0.975–0.982); and 5 = (0.983+). Ranks for vulnerability index: 1 = (0–16); 2 = (16.1–21.5); 3 = (21.6–25.5); 4 = (25.6–31); and 5 = (31.1+). ^2^ PM_2.5_ was standardized by 10. ^a^ *p* < 0.05, ^b^ *p* < 0.01, ^c^ *p* < 0.001. ^†^ *p* = 0.07.

**Table 4:** Number of cardiopulmonary deaths averted annually by reducing PM_2.5_ to low in all census tracts, by high and low vulnerability scores ^1^ under scenarios with 3–15% attributable risk.

Percent Attributable Risk	Low Vulnerability		High Vulnerability	
	Low PM_2.5_	High PM_2.5_	Low PM_2.5_	High PM_2.5_
Population estimates	1,301,007 (30.3%)	657,199 (15.3%)	677,435 (15.8%)	1,659,342 (38.6%)
Cardiopulmonary Mortality Estimates (Total)	1000 (20.8%)	633 (13.2%)	767 (16.0%)	2400 (50.0%)
Attributable to PM_2.5_ (3%)	30 (20.8%)	19 (13.2%)	23 (16.0%)	72 (50%)
Attributable to PM_2.5_ (5%)	50 (20.7%)	32 (13.3%)	39 (16.2%)	120 (49.8%)
Attributable to PM_2.5_ (10%)	100 (20.8%)	64 (13.3%)	77 (16.0%)	239 (49.8%)
Attributable to PM_2.5_ (15%)	150 (20.8%)	96 (13.3%)	116 (16.1%)	359 (49.8%)
Cardiopulmonary deaths averted				
If PM_2.5_ moves from High to Low (3%)		4 (21.1%)		15 (78.9%)
If PM_2.5_ moves from High to Low (5%)		7 (21.9%)		25 (78.1%)
If PM_2.5_ moves from High to Low (10%)		13 (20.6%)		50 (79.4%)
If PM_2.5_ moves from High to Low (15%)		20 (21.1%)		75 (78.9%)

^1^ Low includes census tracts that were in the first and second quintiles of risk; High includes census tracts in the third–fifth quintiles.

## Supplementary Materials

The following are available online at http://www.mdpi.com/1660-4601/15/6/1209/s1. Table S1: Ischemic Heart Disease, Cardiovascular and Cardiopulmonary Mortality Regressed on PM_2.5_ and Population Vulnerability As Continuous Variables, Table S2: Number of cardiopulmonary deaths averted annually by reducing PM_2.5_ to low in all census tracts, by high and low vulnerability scores* under scenarios with 3–15% attributable risk.
