# Supplementary material for: Independent and Joint Contributions of Fine Particulate Matter Exposure and Population Vulnerability to Mortality in the Detroit Metropolitan Area
Source: Int J Environ Res Public Health. 2018 Jun 8;15(6):1209. doi: 10.3390/ijerph15061209 (PMC6024972; doi:10.3390/ijerph15061209)
Supplement: Supplementary file 1 [file ijerph-15-01209-s001.pdf]

Supplemental Tables:

Schulz, A.J., Mentz, G.B., Sampson, N., Ward, M., Dvonch, J.T., DeMajo, R., Israel, B.A., Reyes, A.G., Wilkins, D. Independent and joint contributions of fine particulate matter exposure and population vulnerability to mortality in the Detroit Metropolitan Area.

Supplemental Table 1: Ischemic Heart Disease, Cardiovascular and Cardiopulmonary Mortality Regressed on PM2.5 and Population Vulnerability As Continuous Variables.

	Inschemic Heart Disease			Cardiovascular			Cardiopulmonary		
	Model 1			Model 2			Model 3		
	Odds Ratio	Confidence Interval	*p*-value	Odds Ratio	Confidence Interval	*p*-value	Odds Ratio	Confidence Interval	*p*-value
Intercept	0.21	(0.203,0.217)	<0.001	0.49	(0.473,0.499)	<0.001	0.72	(0.703,0.740)	<0.001
**Level 2 (census tracts)**									
$PM_{2.5}$ (continuous)	1.38	(1.152,1.661)	<0.001	1.11	(0.959,1.291)	0.16	1.22	(1.044,1.416)	0.00
Vulnerability (1-5)	1.03	(0.996,1.064)	0.09	1.03	(1.003,1.055)	0.03	1.04	(1.015,1.065)	0.01

Supplemental Table 2: Number of cardiopulmonary deaths averted annually by reducing PM2.5 to low in all census tracts, by high and low vulnerability scores* under scenarios with 3-15% attributable risk.

Percent Cardiopulmonary Mortality Attributable to PM2.5	PM2.5	Vulnerability	Estimate of Population	Probability of Cardiopulmonary Mortality/Year	Current Cardiopulmonary Mortality/Year Attributable to PM2.5	Cardiopulmonary Mortality/Year Attributable to PM2.5 if High Moves to Low	Cardiopulmonary Deaths Averted/Year if PM2.5 Moves to Low
3%	Low	Low	1,301,007	2.3E-05	30	ref	ref
	Low	High	677,435	3.4E-05	23	ref	ref
	High	Low	657,199	2.9E-05	19	15	4
	High	High	1,659,342	4.3E-05	72	57	15
5%	Low	Low	1,301,007	3.8E-05	50	ref	ref

	Low	High	677,435	5.7E-05	39	ref	ref
	High	Low	657,199	4.9E-05	32	25	7
	High	High	1,659,342	7.2E-05	120	94	25
10%	Low	Low	1,301,007	7.7E-05	100	ref	ref
	Low	High	677,435	1.1E-04	77	ref	ref
	High	Low	657,199	9.7E-05	63	50	13
	High	High	1,659,342	1.4E-04	239	189	50
15%	Low	Low	1,301,007	1.2E-04	150	ref	ref
	Low	High	677,435	1.7E-04	116	ref	ref
	High	Low	657,199	1.5E-04	96	76	20
	High	High	1,659,342	2.2E-04	359	283	75

* Low includes census tracts that were in the 1st and 2nd quintiles of risk, High includes census tracts in the 3rd-5th quintiles
